# Tax Morale of Immigrants

**DOI:** 10.3390/bs16010043

**Published:** 2025-12-24

**Authors:** Nonna Kushnirovich

**Affiliations:** Ruppin Academic Center, Institute for Immigration and Social Integration, Emek Hefer 4025000, Israel; nonna@ruppin.ac.il

**Keywords:** tax morale, immigrants, tax reciprocity, identity, acculturation

## Abstract

This study examined how perceived tax–benefit reciprocity and identification with the country explain tax morale of immigrants versus the native-born population, and how immigrants’ tax morale evolves over time. The study used data from an online survey of 536 people of working age, which were analyzed using Confirmatory Factor Analysis in AMOS, the PROCESS procedure, and ANOVA with Scheffe tests in SPSS ver. 28. The results showed that immigrants have lower tax morale than natives, which is partly explained by their weaker identification with the host country. Low dissatisfaction of immigrants with received benefits versus paid taxes partially buffers the negative relationship between identification and tax morale. After 25 years of residing in the host country, differences in tax morale between immigrants and natives largely disappear, suggesting a gradual process of adjustment and alignment with host society norms. Policy implications of the study suggest that tax morale among immigrants can be improved by strengthening their identification with the host country and enhancing perceptions of fairness in the tax–benefit exchange.

## 1. Introduction

The willingness of citizens to pay taxes is essential for the functioning of society and the well-being of its population. According to economic theory, judgments about paying taxes are made by weighing the trade-off between the benefits of a reduced tax burden and the expenses of penalties for noncompliance (Allingham & Sandmo, 1972). However, many people choose to pay taxes even when chances of an audit and penalties are slight (Torgler, 2007). Scholars explain this by the impact of social elements like morality, societal norms, and views of justice (Cummings et al., 2009; Feld & Frey, 2002; S. X. Li, 2010), or doing the ‘right thing’ (Lubian & Zarri, 2011).

Tax morale is conceptualized in the literature as an intrinsic motivation to pay taxes (Andriani et al., 2022; Cummings et al., 2009) or a moral obligation to pay taxes (Torgler & Schneider, 2007). Numerous studies have shown that tax morale is strongly affected by cultural factors (Andriani et al., 2022; Cummings et al., 2009; Fisman & Miguel, 2007; Fonseca Corona, 2024; Holkova et al., 2023; Kountouris & Remoundou, 2013; Torgler & Schneider, 2007; Torgler, 2004). Despite extensive exploration of the relationship between culture and tax morale, some research niches and gaps remain unaddressed. Fonseca Corona (2024), who reviewed studies on the relationship between tax morale and cultural factors during the past 25 years, found many that offered cross-country analysis, but emphasized a lack of studies examining tax morale from an intra-country perspective. Comparing various groups within the same country sharing a common business environment, public institutions, and legislation may help to better understand the impact of socio-cultural factors, such as identity, on tax morale. Existing studies that examine tax morale among minorities mostly focus on ethnic minorities (Kayaoglu & Wıllıams, 2020; S. X. Li, 2010) rather than immigrant minorities. Although native ethnic minorities and immigrants share some stressors (e.g., discrimination, language differences, lack of local social networks etc.), immigrants face distinctive challenges such as social and economic adjustment in the host country.

The few studies that investigated the tax morale of immigrants (Bastani et al., 2020; Kountouris & Remoundou, 2013; Malézieux & Torgler, 2021; Siddique, 2022) did not address the adjustment of immigrants’ tax attitudes over time, leaving a gap in understanding how integration processes affect motivation for civic behavior such as paying taxes. They also failed to consider the factors that mediate the relationship between immigrant status and tax morale.

A methodological challenge also persists, as most studies on tax morale rely on data from the World Values Survey (WVS), which provides only a single item for assessing tax morale (Brink & Porcano, 2016; Çevik, 2014; Davidescu et al., 2022; Dogan & Secilmis, 2020; Kountouris & Remoundou, 2013; Rutkauskas, 2016; Torgler, 2004, 2006; Torgler & Schneider, 2007). This limited measurement approach may lead to bias in the analysis. Moreover, the WVS item seems more suitable for capturing tax compliance behavior rather than the broader construct of tax morale. In contrast, Lubian and Zarri (2011) proposed a broader framework incorporating multiple dimensions: Kantian, Community, Redistributive, Tax evasion, Tax amnesty, Vertical, and Fairness. Their measurement of tax morale included items addressing perceptions of the unfair distribution of public goods and the misuse of government funds. Arguably, these factors are more relevant as predictors of tax morale rather than measures of tax morale. Thus, a clear need exists for further refinement and development of tax morale measures.

Taken together, there are three main gaps in the literature: existing research rarely examines within-country differences between immigrants and natives, typically relies on single-item measures of tax morale, and largely overlooks how immigrants’ tax attitudes adjust over time. To address these gaps, this study investigates how perceived tax–benefit reciprocity and identification with the country explain the tax morale of immigrants vs. the native-born population, and how immigrants’ tax morale evolves over time in the host country. In this study, an ‘immigrant’ was defined according to the definition of the Statistics Division of the United Nations Department of Economic and Social Affairs (UN DESA, 1998, p. 10): “A person who moves to a country other than that of his or her usual residence for a period of at least a year (12 months), so that the country of destination effectively becomes his or her new country of usual residence”.

This study makes several contributions to the literature on tax morale and immigrant integration. First, it shows that the tax morale of majority and minority groups may differ even though they operate within an intranational context (e.g., legislation, public institutions, and the business environment of the same country). Second, unlike prior research, this study uses a multi-item construct of tax morale, offering a more nuanced measure. Third, the study’s novelty lies in identifying two new mediators of the relationship between being an immigrant and tax morale: a mediator of feelings of dissatisfaction with the benefits received in return for tax payments, and a mediator of identification with the country. Fourth, the study explains how immigrants’ tax morale changes over time in the host country, which is regarded as a proxy for immigrants’ business ethics acculturation. The findings highlight the importance of both integration and institutional trust-building for enhancing tax morale in diverse societies.

The paper is organized as follows: it opens with a theoretical background and a literature review, after which the study’s conceptual model is formulated. This is followed by an explanation of the method and results. The paper concludes with a discussion of the study’s findings.

## 2. Theoretical Background

### 2.1. Tax Morale of Immigrants vs. Natives

Previous studies found differences in tax compliance and tax morale between immigrants and natives (Bastani et al., 2020; Kountouris & Remoundou, 2013; Malézieux & Torgler, 2021). For instance, foreigners, who constitute only six percent of France’s population, account for 23 percent of the country’s tax evasion charges (Spire & Weidenfeld, 2015). In Sweden, immigrants not only claim fewer deductions but are also more likely to miss declaration deadlines and face fines for noncompliance (Bastani et al., 2020). In Israel, Al-Haj and Leshem (2000) found notable disparities in attitudes about white-collar crime, where immigrants from the Former Soviet Union (FSU) demonstrated a greater tolerance for white-collar crimes than did natives.

Since immigrants and natives of a given country share the same institutional and economic context (legislation, business environment, and public institutions), variations in tax morale can be at least partially attributable to differences in the national culture and social norms (Kountouris & Remoundou, 2013). The impact of cultural and social norms on tax morale has been extensively investigated (Andriani et al., 2022; Cummings et al., 2009; Fisman & Miguel, 2007; Fonseca Corona, 2024; Holkova et al., 2023; Kountouris & Remoundou, 2013; Torgler, 2006). It is especially important for immigrants who may have internalized tax-related norms and behaviors from their countries of origin (Fisman & Miguel, 2007; Kountouris & Remoundou, 2013). Indeed, previous empirical studies found that cultural and social norms of the country of origin have an impact on the perceptions of corruption and the behavior of immigrants (Fisman & Miguel, 2007; Kountouris & Remoundou, 2013). Usually, immigrants from low (high) tax morale countries are less (more) compliant than natives (Kountouris & Remoundou, 2013; Malézieux & Torgler, 2021). Siddique (2022) called this phenomenon a ‘sticky effect of corruption’. People from countries with systemic corruption have less faith in public officials, and their tax morale declines because they feel defrauded (Torgler, 2007). In such countries, tax evasion is a self-defense tactic employed by citizens (Horodnic, 2018). At the opposite end of the scale, immigrants from countries with higher tax morale reported being more morally inclined to pay taxes in the host countries (Kountouris & Remoundou, 2013).

Disparities in tax morale between natives and immigrants may not be fully explained by cultural differences. They may also result from intergroup biases, political and economic disadvantages, and discrimination (Siddique, 2022; S. X. Li, 2010). Immigrants are more likely than natives to be politically disadvantaged; they are usually less involved in political activities and do not believe they can influence government policy (R. Li & Jones, 2020). Intergroup bias emerges when a native-born group is the primary beneficiary of public goods and public programs from which immigrants are either totally or partially excluded. Youngmann and Kushnirovich (2020) found that perceived levels of common unfairness in public institutions impinged upon the well-being of immigrants to a similar extent as personally experienced discrimination. Due to economic and political disadvantages, immigrants may feel that they have not been treated fairly and report poorer tax morale (Siddique, 2022). Moreover, immigrants may justify their low tax morale by their bad economic situation due to immigration, and by a need to survive (Malézieux & Torgler, 2021). All these may contribute to differences in tax morale between natives and immigrants.

Based on the literature review, Hypothesis H1 can be formulated as follows:

**H1.**  *Being an immigrant will be negatively associated with tax morale.*

### 2.2. Tax–Benefit Reciprocity and Tax Morale

The psychological foundations of incentives formulated by Fehr and Falk (2002) distinguish among several basic motives for economic incentives: the motive to reciprocate, the desire for social approval and maintenance of social norms, and the desire to work on interesting tasks. Incentives based on reciprocity are the most pertinent and powerful economic motivations (Fehr & Falk, 2002). In the context of public goods, reciprocity may be regarded as a form of “conditional cooperation” (Fehr & Falk, 2002), meaning that people are willing to contribute when others also contribute—the basic principle underlying tax payment.

Studies ground incentives to pay taxes in the Kantian approach to morality (Lubian & Zarri, 2011), where the perceived fairness of the tax burden depends on the quantity and quality of public goods provided by the state (Castañeda, 2024; Castañeda-Rodríguez, 2025; Doerrenberg & Peichl, 2022); namely, on tax–benefit reciprocity. In line with this, Cummings et al. (2009) found that tax compliance was higher when government services were perceived as fair, transparent, and generally desirable. When taxpayers feel that the state fails to provide adequate services in return for taxes, their intrinsic motivation to comply (tax morale) declines. In this sense, tax morale may be viewed as an implicit or “psychological” contract between individuals and their government, whereby people expect to receive public goods and services in return for paying taxes (Feld & Frey, 2002).

The Tax Compliance Model (Allingham & Sandmo, 1972; Yitzhaki, 1987), which is widely used in studies on tax compliance and tax morale, posits that tax compliance decisions are based on the trade-off between the benefits gained from tax evasion and the penalties for noncompliance. According to this model, benefits received in return for taxes are an important determinant of paying taxes (Cummings et al., 2009; Giaccobasso et al., 2025; Lubian & Zarri, 2011). The public’s confidence in tax reciprocity is the primary factor for tax morale (Fotiadis & Chatzoglou, 2021). tax–benefit reciprocity between the state and citizens may be regarded as a rational motivation for tax morale because it is based on taxpayers’ evaluation of the material returns from the tax paid, i.e., on exchange-based, benefit-driven calculus (Gribnau & Dijkstra, 2019).

Empirical research has demonstrated that the tax morale of people who are likely to receive lower benefits from the welfare state, such as the self-employed (Alm et al., 2006; Torgler, 2004) and high-income earners (Torgler, 2006; Alm et al., 2006), is lower than that of other groups (Rodriguez-Justicia & Theilen, 2022). For high-income earners, tax progressivity plays a role (Hoy, 2025). Benefits from paying taxes also include the quality of public goods and services provided (Feld & Frey, 2002), which may be perceived differently according to ethnic fractionalization (Lago-Peñas & Lago-Peñas, 2010).

Tax morale increases among individuals who are confident that the public authority acts in the interest of tax contributors (Feld & Frey, 2002). Majority groups, which consume most public programs, are more altruistic and cooperative than minority immigrants; they also have higher tax morale (Siddique, 2022). The moral cost of tax evasion falls if the state is seen as having little commitment to providing public services. According to Frey and Torgler (2007), residents may therefore feel less motivated to fulfill their responsibilities, such as paying taxes, and be less worried about facing consequences for their tax evasion behavior (Andriani et al., 2022).

Thus, if people believe that personally or as an ethnic or social group they will benefit because they pay taxes, their tax morale will be higher. For immigrants who benefit less from the public system, this factor may be important for shaping their tax morale. Based on this, the following hypotheses may be formulated:

**H2.**  *Being an immigrant will be positively associated with feelings of dissatisfaction with the benefits received in return for tax payments.*

**H3.**  *Feelings of dissatisfaction with the benefits received in return for tax payments will be negatively associated with tax morale.*

### 2.3. Identification with the Country and Tax Morale

The desire for social approval (or to avoid social disapproval) and the maintenance of social norms are additional psychological foundations of economic incentives (Fehr & Falk, 2002). Social norms may shape not only social behavior but also individuals’ economic behavior (Torgler, 2004). Bobek et al. (2013) found that the effects of personal subjective social norms on tax behavior are stronger than the effects of detection risk and perceived fairness. Social norms are therefore a salient psychological factor for tax compliance and tax morale (Bobek et al., 2013). Cialdini and Trost (1998) included within social norms both external social rules and standards, as well as a person’s own moral or ethical convictions. Immigrants may hold mixed social norms when their moral convictions reflect those of their country of origin, while their adjustment to the host country also exposes them to the external social norms of the host society and business environment (Jaffe et al., 2018).

People’s adoption of a nation’s shared norms is reflected in their identification with the national community (Tyler & Blader, 2003). When individuals strongly identify with their country, they are more likely to perceive compliance with collective rules, including the payment of taxes, as a means of sustaining the social order to which they belong. Identification with the country and nation functions as a mechanism for maintaining social norms, reinforcing adherence to behaviors regarded as legitimate and morally expected within the community (Feld & Frey, 2007). As such, identification with the country may be regarded as a normative motivation that reflects adherence to moral and social norms that go beyond a material or transactional calculus or the pursuit of rewards (Kelly & Davis, 2018).

Studies have shown that identification with the state, the nation, or the country is strongly associated with tax morale (Martinez-Vazquez & Torgler, 2009). S. X. Li (2010) found that personal tax morale was influenced both by ‘external’ identities based on externally observable ethnic, linguistic, or religious characteristics, and by ‘internal’ identity based on a self-reported sense of citizenship. People who considered themselves citizens had higher tax morale than those who did not, whereas the effect of such identification on tax morale depended on the extent of ethnic fragmentation of the nation (S. X. Li, 2010). Similarly, Mickiewicz et al. (2019) found that a greater feeling of affiliation to the nation was associated with higher tax morale. While national pride (Konrad & Qari, 2012; Kountouris & Remoundou, 2013) and patriotism (Fonseca Corona, 2024; Rodriguez-Justicia & Theilen, 2022) are associated with higher tax morale, contempt for one’s own nation is associated with low tax morale (Rutkauskas, 2016).

Host country identification is generally lower among immigrants compared to natives, with some variation between immigrant groups (de Vroome et al., 2014). Moreover, economic and political disadvantages experienced by immigrants may lead to their social exclusion. Based on this, difficulties in the new country may result in lower tax morale even among immigrants who came from countries with high tax compliance.

Nesdale and Mak (2000) stressed the importance of distinguishing between ethnic identity and identification with the host country, where the latter is “mainly determined by a more explicit and pro-active attitude of wanting to fit into, and live according to, the norms and standards prevailing in the host culture” (p. 485). Kayaoglu and Wıllıams (2020) showed that individuals whose main identity was based on their citizenship (in this case Turkey) reported higher tax morale than those whose main identity was based on their ethnicity. Correspondingly, two hypotheses may be formulated:

**H4.**  *Being an immigrant will be negatively associated with identification with the host country.*

**H5.**  *Identification with the host country will be positively related to tax morale.*

### 2.4. Link Between Tax Morale and Immigrants’ Acculturation

According to the Cultural Stress Theory (CST) (Meca & Schwartz, 2024), immigrants deal with stressors by developing a host country identity and identifying with it, under the impact of the business, law, and tax environment in the host country. Over time, they adjust and come closer to the local population. Immigrants may implement one of several strategies to acculturate: assimilation (accepting cultural values and social norms of the host country and rejecting those of the home country), integration (accepting values of both host and home country), separation (rejecting values of the host country and maintaining values of the home country), marginalization (rejecting values of both host and home country) (Berry, 2005).

Dis/satisfaction with the public benefits and goods received in return for tax payments can depend on the acculturation strategy applied by immigrants. For example, an integration strategy, which is associated with bicultural identity, heightens institutional trust and greater internalization of civic norms of the host country, which, in turn, may elevate the tax morale of immigrants. Dong et al. (2025), who extended Berry’s Acculturation theory to the case of internal migrants in China, found that migrants who applied integration or assimilation strategies had a higher level of trust in healthcare, which may be regarded as a public good, compared to those who adopted a separation strategy. However, Wenzel (2006) found that Hispanic immigrants in the USA who preferred social communication with natives and used the language of the host country in daily life, which corresponds with integration and assimilation strategies, reported lower trust in both local and central government and higher dissatisfaction with their activities. Trust in government organizations that provide public goods and services can significantly impact tax morale (Koumpias et al., 2020). Immigrants who apply integration and assimilation strategies demonstrate higher national identification with the host country compared to those who adopt separation and marginalization strategies (Verkuyten & Martinovic, 2012). This host-country identification may, in turn, enhance tax morale. Thus, integration and assimilation strategies expose immigrants to the host society’s civic norms and institutions, reinforcing perceptions of fair exchange and shared belonging, which may elevate tax morale. In contrast, separation or marginalization reduce contact with society and institutions, limiting both perceived reciprocity and national identification, thereby lowering the moral motivation to comply.

Another concept of segmented assimilation posits that immigrant groups may assimilate into only a limited number of social fields of those available in the host society (Portes & Zhou, 1993). Segmented assimilation, where immigrants participate in certain host-society domains (e.g., labor market, education) but remain excluded from others (e.g., civic and political life), can create mixed effects on tax morale. Economic integration increases exposure to formal institutions and fiscal obligations, fostering compliance. Yet exclusion from welfare and political participation weakens perceptions of reciprocity and identification with the state, reducing intrinsic motivation to contribute. In their study on the acculturation of immigrant youth in Israel, Berry et al. (2006) found that co-ethnic contacts, that is, maintaining one’s cultural heritage, had an impact on psychological adaptation but not on sociocultural adaptation, which is relevant to adopting social norms such as paying taxes. Thus, even if one applies a separation strategy in some domains of their life, s/he can be integrated in other domains (for example, in the labor market) and have higher tax morale. On the other hand, segmented assimilation produces partial belonging and identification (Lopez et al., 2021; Portes & Zhou, 2025), and low identification can lead to lower tax morale. Segmented assimilation shows that partial inclusion, such as economic integration without civic engagement, can produce conflicting outcomes by fusing formal compliance with a lack of internal commitment.

Acculturation frameworks help explain why immigrants differ in both their dis/satisfaction with tax reciprocity and their identification with the host country, two mechanisms underlying immigrants’ business ethics and tax morale. Some scholars note parallels between the acculturation of immigrants in their host society and the process of adjusting their business ethical codes to those of the new country (Bashe et al., 2007; Handelsman et al., 2005). When immigrants adapt to the new business environment, they adopt a set of ethical rules of behavior that become part of their business culture (Jaffe et al., 2018). For example, McDonald and Pak (1996) found that Hong Kong managers residing in Canada developed a unique set of ethical beliefs that differed from those of local managers in Hong Kong and also from the beliefs of local Canadian managers. In Israel, FSU immigrants’ tolerance for white-collar crime was correlated with their level of embeddedness in the host culture and culture of origin: the more integrated they were into Russian culture and the more cut off from the society of the host country, the more accepting they were of white-collar crime (Leshem & Neeman-Haviv, 2013). Jaffe et al. (2018) showed that immigrants combined business ethical beliefs based on both home and host country cultures, but over time, their business ethics beliefs tended more towards those of natives. According to Hedegaard (2023), migrants adapt their attitudes towards the tax burden distribution to those of the native population, but this process requires time.

Correspondingly, it may be hypothesized that:

**H6.**  *The more years immigrants reside in the host country, the more their level of tax morale will resemble that of natives.*

## 3. Method

### 3.1. The Model

**Figure 1:**
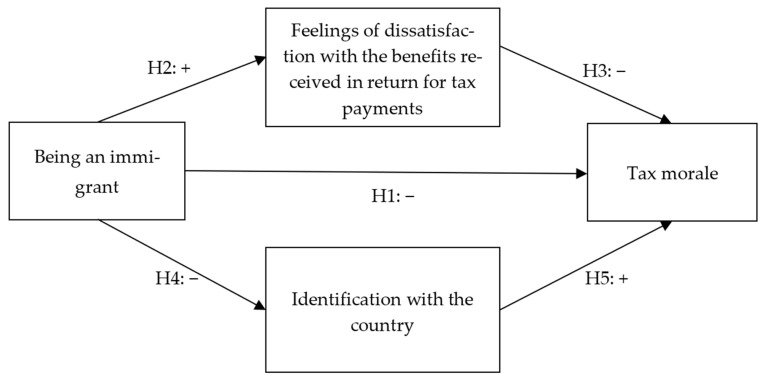
Conceptual model of the study.

Based on the literature review, the model of this study is as follows. Tax morale is predicted by the fact of being an immigrant, namely, by carrying perceptions of the country of origin and the immigrant experience in the host country. Tax morale is also predicted by the following considerations: dissatisfaction with tax–benefit reciprocity would predict it negatively, and identification with the country would predict it positively. Being an immigrant is negatively related to identifying with the host country and dissatisfaction with the benefits received in return for tax payments. The conceptual model of the study is presented in Figure 1.

Demographic and socio-economic characteristics also play a role. Age is positively associated with tax morale, whereas being self-employed or not working is negatively associated (Lubian & Zarri, 2011; Rodriguez-Justicia & Theilen, 2022). Women have higher tax morale than men (Lubian & Zarri, 2011). Regarding education, some studies found a positive association between it and tax morale (Lubian & Zarri, 2011), and others found no association (Rodriguez-Justicia & Theilen, 2022).

A particular determinant that predicts the tax morale of immigrants is years of residence in the host country, which may be regarded as a proxy for immigrants’ acculturation (Jaffe et al., 2018).

As the literature review showed, tax morale is a multidimensional concept that needs broad and generalized measuring. Many empirical studies on tax morale rely on data from the European and World Values Surveys (EVS and WVS), which include one question on tax cheating justifiability (Çevik, 2014; Davidescu et al., 2022; Dogan & Secilmis, 2020; Kountouris & Remoundou, 2013; Martinez-Vazquez & Torgler, 2009; Malézieux & Torgler, 2021; Rodriguez-Justicia & Theilen, 2022; Torgler & Schneider, 2007; S. X. Li, 2010). They employ a single-item measure that corresponds more closely to tax compliance than to tax morale. Such a rough measurement may result in analytical bias. As Marien and Hooghe (2011) showed, asking direct questions on deviant behavior may cause biased results since respondents are reluctant to report it. The scholars who used the single-item measure of WVS themselves stressed that tax morale is a multidimensional concept, which needs a multi-item measurement tool, and that a multi-item index would be less likely to be negatively impacted by random errors, thus providing a more accurate measurement (Torgler & Schneider, 2007).

Lubian and Zarri (2011) examined tax morale using a set of questions that represented several dimensions: Kantian (paying taxes is a basic duty of citizenship), Community (not paying taxes is a crime), Redistributive (taxes help the weak), Tax evasion (the government’s problem), Tax amnesty (opinion on amnesties), Vertical motivation (how the government spends money), and Fairness (you may not pay unfair taxes), but they did not unite them into one aggregated index. Torgler (2004) created an index of tax morale, which was based on 10 items and included dimensions of both economic survival (‘Tax evasion is an economic necessity for many to survive’) and normalization of tax evasion as a common and widespread phenomenon (‘Everybody evades taxes’, ‘There is nothing bad about under-reporting taxable income’). However, he did not explain whether or how this index was validated. Another aggregate index of tax morale was proposed by Taing and Chang (2021). This index, even validated statistically, included items reflecting only a limited number of aspects of tax morale, such as willingness to report liabilities correctly and not evade taxes (4 items), perceiving taxes as an obligation (1 item), and a general judgment of evading taxes as something wrong (1 item). However, it did not reflect the entire spectrum of tax morale’s dimensions, omitting beliefs about the harm to society by tax evaders, legitimatization and normalization of tax evasion, and presenting it as a choice compelled by circumstances.

Based on the multidimensional nature of tax morale encompassing beliefs about community fairness, government integrity, and moral justifications for evasion, this study conceptualizes tax morale as a generalized moral attitude toward taxation, that is, an overarching construct integrating these evaluative components. It broadened the standard measurement to include both negative and justificatory aspects of tax morale, thereby covering the full evaluative continuum from moral condemnation of evasion to its normalization. Theoretical precedents support this integrative view: Torgler (2004), Frey and Torgler (2007), and Malézieux and Torgler (2021) all modelled tax morale as a general moral attitude, akin to ‘civic virtue’ or ‘public spirit.’ A broader construct captures moral commitment to taxation in its full spectrum, rather than focusing on a single moral aspect.

### 3.2. Data and Sample

This research is based on an online survey of 536 employed respondents and potential taxpayers, administered in Israel in two steps: in 2022 and in 2024. The decision to proceed to the second step in 2024 was made because of the commencement of the war between Israel and Hamas on 7 October 2023, which might have led to changes in tax morale. Due to the war, the government faced fiscal challenges, which led to a 1 percent increase in the VAT rate. The population experienced threats and losses to its security, income, and other resources. In times of national crises, socially vulnerable groups like immigrants are the most susceptible to the losses (Blukacz et al., 2023), which may change their tax morale. However, conducting the survey in two steps might pose potential risks for the robustness and reliability of the empirical results. To mitigate possible biases and control the effect of the survey’s steps, a dichotomous control variable for ‘year’ was included in the data analysis. No differences in tax morale between the steps were found (see Section 4).

Creating a representative sample of migrants is always challenging because of the low willingness of vulnerable population and immigrant groups to participate, and the feeling of threat they experience (Markova et al., 2020). To reach less accessible populations (Heckathorn, 2011), such as ‘hard-to-reach’ immigrants, especially in times of crises or vulnerabilities (Blukacz et al., 2023), the snowball sampling method is highly recommended. To obtain accurate quantitative data when populations are difficult to reach, snowball sampling is crucial or the only method (Ting et al., 2025). In case of not reaching the population with diverse characteristics by snowball sampling, Blukacz et al. (2023) recommended complementing snowball sampling with other sampling methods. Furthermore, respondents perceive questions about tax avoidance as delicate or risky, that might strengthen a sense of threat during a survey. In such cases, snowball and convenience sampling, which can foster trust between the interviewer and interviewees and help moderate a potentially unpleasant survey experience, are recommended (Cohen & Arieli, 2011). The mix of snowball and convenience sampling methods increases the likelihood of involvement for vulnerable immigrant groups, who may be reluctant to do so because of language problems and mistrust of authorities and the local population (Markova et al., 2020). This study used a combination of snowball and convenience sampling methods.

At each stage (2022 and 2024), fifteen research assistants received a link to the online questionnaire and were instructed to collect responses from about twenty participants each, keeping variance in gender, age, and geographic location, which were based on the demographic characteristics of the Israeli population according to the Central Bureau of Statistics of Israel. Most of the research assistants were BA students. For the purposes of the study, and based on the definition of UN DESA (1998) mentioned in the Section 1, immigrants were regarded as foreign-born individuals who moved to Israel and resided there for a period of at least 12 months. To examine how immigrants’ tax morale evolves over time in the host country, the research assistants were instructed to reach immigrants with both short and long tenures in Israel. Responses with missing data were excluded from the survey. To maintain the anonymity promised to interviewees, the database did not include respondents’ email addresses, telephone numbers, or other details.

The population in Israel is relatively young; according to the Central Bureau of Statistics of Israel, 56% of it is under 35 years old (Central Bureau of Statistics of Israel, 2023). Among the adult population (18+), the largest group is young people aged 18–19 (4.2% of the adult population). The immigrant population in Israel is also young, but slightly older than the native-born people: in 2022, 48.6% of immigrants to Israel were under 35 years old (Ministry of Aliyah and Integration of Israel, 2025). The Israeli population is educated, but immigrants who come to Israel are even more educated: 37.2% of native-born persons hold an academic diploma (Central Bureau of Statistics of Israel, 2023), compared to 67.1% of immigrants who worked before immigration in an academic occupation. About 3 percent of them hold Ph.D. or equivalent degrees compared to 1.1 percent of the native population (Central Bureau of Statistics of Israel, 2024). In 2022, the average wage per employee in Israel was 11,766 ILS (3362 USD).

The characteristics of the study’s sample are presented in Table 1. Of the 536 respondents, 389 were natives and 147 were immigrants. Fifty-nine percent were surveyed in 2024, and 41 percent in 2022. About half of each group were male. The average age of participants was 33.9 (SD = 13.0) for the native-born population and 38.3 (SD = 14.9) for immigrants. It seems that the study’s sample is slightly younger than the national population described above. However, this study did not aim to provide a representative sample, as the survey strategy was based on snowball and convenience sampling. The primary objective was to ensure that the sample encompassed respondents with diverse characteristics by age, gender, education, etc., and reflected broader demographic trends observed in the host society, in particular that immigrants are older and more highly educated than the native-born population.

In the study’s sample, immigrants were more educated than the native-born group (59.2% of them held an academic degree vs. 44.7% of the native-born persons). This gap is in line with the national statistics described above. The average income of respondents (all of them were employed) corresponded to the average wage in Israel in 2022, with a higher income among native-born persons. This reflects the situation in Israel, where studies found a gap between the wages of the native-born and immigrant populations (Chiswick et al., 2016; Semyonov et al., 2015). In the sample, 57 percent of the immigrants were from the FSU (44 percent from Russia and Ukraine), 23 percent from Europe, 13 percent from the Americas, and 7 percent from Asia and Africa. This matches national figures showing that over half of Israel’s immigrants during the past 35 years are from the FSU. Twenty-six percent of immigrants had resided in Israel at the time of the survey for less than 10 years, 35 percent for 11–25 years, and 39 percent for 26 or more years. Previous studies used a similar threshold by tenure in the host country: immigrants who have resided in the host country for less than 10 years (Brunow & Jost, 2022; Röder & Mühlau, 2014) and more than 25 years (de Palo et al., 2006; Röder & Mühlau, 2014), when the latter was defined as a very long time of stay (de Palo et al., 2006). 25.7 percent of the native-born persons were entrepreneurs vs. 22.4 percent of immigrants (the others were salaried workers).

### 3.3. Variables

Dependent variable. For measuring tax morale, broad and multidimensional measure was developed based on nine items scaled 1–5 (see Table 2). These nine items were integrated into a one-factor index. This integration is justified by the idea that people’s condemnation of evasion (e.g., ‘tax evaders hurt us all’) and their justifications (e.g., ‘sometimes there is no choice’) may be perceived as two poles of the same continuum of moral approval versus disapproval of tax cheating. Treating them as separate subdimensions might fragment what is, in practice, a single latent orientation toward the moral obligation to pay taxes. The broadened nine-item construct was designed to capture the full continuum from moral condemnation of tax evasion to its partial legitimation, reflecting both positive and negative evaluations of tax behavior within a single attitudinal framework. As Frey and Torgler (2007) argued, creating a multi-item unified index of tax morale is preferable because such an index is less likely to be adversely affected by random errors.

The measure of tax morale was justified by Confirmatory Factor Analysis (CFA) run in AMOS 28. The fit indices of the model were good (*CFI* = 0.986, *NFI* = 0.974, *IFI* = 0.986, *TLI* = 0.970, *RMSEA* = 0.045). Alternative models with three subfactors (normalization of tax evasion, tax evasion as a crime, and evasion as a harm to society) and two subfactors (normalization of tax evasion and paying taxes as a duty) were tested (Appendix A, Table A1). The fit indices for these models were worse than those for the single-factor model (*CFI* = 0.974, *RMSEA* = 0.067 for the three-factor model; *CFI* = 0.968, *RMSEA* = 0.065 for the two-factor model). In line with the core psychometric principle of parsimony, when a simpler model fits well, it is preferred over more complex alternatives (Bollen, 1989).

One-, two, and three-factor models are distinct from one another not by added constraints but by different factor structure; thus, they are not-nested within one another. To compare non-nested models, the information criterions *AIC* and *BIC* were examined, as advised by Browne and Cudeck (1992); model fit is better when the *AIC* and *BIC* values are smaller (Merkle et al., 2016). Both *AIC* and *BIC* for the one-factor model had smaller values than those of the alternative two-factor model and three-factor model (Appendix A, Table A1). Models may be considered different if there is a gap in *BIC* of 10 points or more (Raftery, 1995). In this study, the BIC difference between the one- and two-factor models was 36.328, and between the one- and three-factor models 22.685, which is more than 10 points. Thus, the one-factor model was significantly different from the two- and three-factor models and provided the best fit to the data. These results justify the interpretation that respondents perceived all nine items as expressions of one underlying moral disposition toward taxation. The internal reliability of the index was acceptable (Cronbach’s alpha = 0.832). Together, these findings support the use of a unidimensional measure, representing the most parsimonious and empirically supported operationalization of tax morale while remaining consistent with the literature.

Independent variables. Feelings of dissatisfaction with the benefits received in return for tax payments was presented by a single-item variable based on the question “I feel that I pay a high tax compared to what the government provides me”, scaled 1–5, from 1 = do not agree at all, to 5 = completely agree. Identification with the country was based on the question “To what extent do you feel your identity aligns with Israel”, scaled 1–5, from 1 = to a very small extent, to 5 = to a very high extent.

Because the war that had begun in Israel in 2023 might impact the tax morale of the participants, a dichotomous control variable for Year was included, coded 1 = 2024 and 0 = 2022.

The variables are presented in Table 2.

For the analysis, the PROCESS procedure in SPSS ver. 28 and CFA in AMOS ver. 28 were used. The analysis controlled for age, gender, education, income, and other characteristics of the sample, and then the predicted values of tax morale were compared between the groups. Since the sample of the study was non-representative and non-probabilistic, predicted values of the tax morale received from regressions were used for data analysis, while controlling for demographic and socioeconomic characteristics of the respondents.

## 4. Results

### 4.1. Examining Determinants of Tax Morale

The index of tax morale was *M* = 3.34 (*SD* = 0.69) on a scale from 1 to 5, indicating moderate tax morale. Feelings of dissatisfaction with the benefits received in return for tax payments and identification with the country were rather high (*M* = 4.20, *SD* = 0.96, and *M* = 4.13, *SD* = 1.10, correspondingly, on a scale from 1 to 5). The mean score of 4.2 on dissatisfaction reflects the reality in Israel, where dissatisfaction with government policy is relatively high. In 2023, the protest movement began in response to judicial reform proposals and resurfaced during the Israel–Hamas war (Ganel, 2024; Navot & Goldshmidt, 2025). Demonstrators voiced strong discontent with budget allocations and with other issues related to the war.

To examine hypotheses 1–3, the PROCESS Procedure, Version 4.2 for SPSS (Preacher & Hayes, 2004) was used. The standard Model 4 of the PROCESS procedure, which is used for analyzing mediation effects of one or a few mediators when using multiple covariates, was run. It included three regressions examining mediation: two regressions predicting mediators, and one regression where the independent variables were being an immigrant, two mediators and covariates, and the dependent variable was tax morale. The model included two mediators: feelings of dissatisfaction with the benefits received in return for tax payments and identification with the country. The covariates were sex, age, education, household income, being an entrepreneur, and year of survey. Multicollinearity among the independent variables was examined using variance inflation factors (VIF). As a rule, a VIF above 5 (or above 2.5 under very conservative standards) indicates a multicollinearity problem. In this analysis, all VIFs were below 1.702. Therefore, no multicollinearity problems were detected in the model.

**Figure 2:**
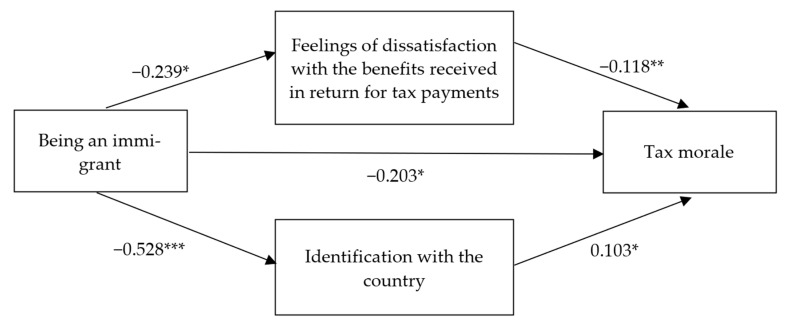
Standardized effects of the model. Indirect effects of being an immigrant on tax morale through feelings of dissatisfaction with the benefits received in return for tax payments: *β* = 0.028, *LLCI* = 0.003, *ULCI* = 0.066. Indirect effects of being an immigrant on tax morale through identification with the country: *β* = 0.054, *LLCI* = −0.108, *ULCI* = −0.011. Total effect of being an immigrant on tax morale: *β* = −0.229, *p* = 0.016, *LLCI* = −0.2978, ULCI = −0.0304. *** *p* < 0.001; ** *p* < 0.010; * *p* < 0.050.

The standardized effects of the model are shown in Figure 2. The model tests whether the observed covariance structure is consistent with theoretically grounded hypotheses linking immigrant status, identification, dissatisfaction, and tax morale. Although causal paths cannot be established based on cross-sectional, convenience-sampled data, in structural equation modeling the estimated paths represent theoretically specified directional relationships rather than empirically verified causal effects. Nevertheless, the study uses terms such as ‘effect’, which is common practice in the SEM literature to describe path coefficients and mediated relationships (Bollen, 1989).

The study found that among respondents of the survey, being an immigrant was negatively associated with tax morale (direct effect *β* = −0.203, *p* = 0.037, and total effect *β* = −0.229, *p* = 0.016), namely, the tax morale of immigrants was significantly lower than that of natives. Hypothesis H1 was supported.

Being an immigrant was negatively associated with feelings of dissatisfaction with the benefits received in return for tax payments (*β* = −0.239, *p* = 0.015). In other words, immigrants were less likely to be dissatisfied with the benefits they received in return for the taxes they paid than natives. Hypothesis H2 was not supported. Feelings of dissatisfaction with the benefits received in return for tax payments, in turn, were negatively related to tax morale (*β* = −0.118, *p* = 0.005). Thus, dissatisfaction with the benefits vs. paid taxes was associated with lower tax morale. Hypothesis 3 was supported. The indirect effect of being an immigrant on tax morale through feelings of dissatisfaction with the benefits received in return for tax payments was positive and significant (*β* = 0.028, *LLCI* = 0.003, *ULCI* = 0.066); thus, dissatisfaction with benefits mediated the relationship between being an immigrant and tax morale. It is important to note that these findings pertain specifically to the sample of respondents included in this study.

Being an immigrant respondent was negatively associated with identification with the country (*β* = −0.528, *p* < 0.001). Hypothesis H4 was supported. In turn, identification with the country was positively associated with tax morale (*β* = 0.103, *p* = 0.017). Hypothesis 5 was supported for the sample of the study. The indirect effect of being an immigrant on tax morale through identification with the country was negative and significant (*β* = −0.054, *LLCI* = −0.108, *ULCI* = −0.011). Thus, identification with the country also mediated the relationship between being an immigrant and tax morale.

The full list of standardized effects of the mediation model is presented in Table 3. All regressions were significant at the level of *p* < 0.001. Among the sample respondents, people with higher incomes reported higher feelings of dissatisfaction with the benefits received in return for their taxes, but highly educated people reported lower dissatisfaction. Being male was negatively related to identification with the country, and income was positively related to identification. Tax morale was positively predicted by age and income, and was negatively predicted by being an entrepreneur. Thus, the tax morale of older and high-income people was higher, and the tax morale of entrepreneurs was lower than that of other people.

### 4.2. Adjustment of Immigrants’ Tax Morale over Time Residing in the Host Country

To examine how tax morale changes over time, an ANOVA with Scheffe tests between the groups was run. The categorical measures for time were chosen because of the relatively small number of respondents.

The differences in tax morale between the groups are presented in Table 4. The table shows that the tax morale of immigrant respondents who have resided in Israel for less than 10 years (*M* = 3.21, *SD* = 0.021) was similar to that of immigrants who have resided there for 11–25 years (*M* = 3.19, *SD* = 0.22), the *Mean difference* = 0.023, *p* = 0.969. However, the tax morale of both groups was significantly lower than that of immigrant respondents who have resided in Israel for 26 years or more (*M* = 3.45, *SD* = 0.27), and significantly lower than the tax morale of natives (*M* = 3.40, *SD* = 0.023). No significant *Mean difference* in tax morale was found between immigrants who have resided in Israel for 26 years or more and natives (*Mean difference* = 0.054, *p* = 0.469). Thus, in regard to tax morale, immigrants who have resided in Israel for a long time (26 years or more) were more similar to natives than to other immigrants who have resided in Israel for less than 25 years (less than 10 years and 11–25 years). Hypothesis H6, which states that the more years immigrants reside in the host country, the more their level of tax morale will resemble that of the natives, was supported for the study’s sample. After 25 years of residing in the host country, the tax morale of immigrants has fully adjusted to that of natives.

Immigrant respondents of the sample, who have resided in Israel for less than 10 years reported the lowest level of feelings of dissatisfaction with the benefits received in return for tax payments (*M* = 3.15, *SD* = 1.21); this level was significantly lower than for all other groups. Namely, immigrants who have resided in Israel for less than 10 years were the most satisfied with the benefits they received vs. the taxes they paid. Feelings of dissatisfaction with the benefits received in return for tax payments increased over time of residence in the host country. No differences were found between immigrants who have resided in Israel for 11–25 years (*M* = 4.42, *SD* = 0.086), 26 years and more (*M* = 4.20, *SD* = 0.91), and natives (*M* = 4.29, *SD* = 0.87).

Immigrant respondents who resided in Israel for less than 10 years reported the lowest identification with the country (*M* = 3.54, *SD* = 1.07), with significant differences from all other groups. Over time of residence in the host country, identification with the country rose. For immigrants who have resided in Israel for 10–25 years *M* = 4.06, *SD* = 1.04, which was higher than for immigrants who have resided there for less than 10 years, but still lower than the identification of natives (*M* = 4.50, *SD* = 0.78). For immigrants who have resided there 26 years or more, identification with the country (*M* = 4.44, *SD* = 0.88) was similar to that of natives (no difference was found between these two groups).

## 5. Discussion

This study explained how rational motivation, in terms of feeling that the government provides satisfactory benefits in return for paid taxes, and social-psychological motivation, in terms of identification with the country, relate to tax morale of immigrants and natives in the examined sample in Israel. This study offers new insights into how both kinds of motivations explain tax morale among immigrants and natives from an intra-national perspective of one country. The study’s novelty lies in revealing two new mediators: dissatisfaction with tax–benefit reciprocity and identification with the country, and in showing how the gap in tax morale closes over time, with gaps in tax–benefit reciprocity closing faster than the gaps in identification. This study also develops a new construct of tax morale, in contrast to most previous studies, most of which were based on a single-item measure or on a few separate dimensions. It contributes to both the business ethics and migration literature.

The study found that the tax morale of immigrants in the examined sample was significantly lower than that of natives. This can be explained in several ways in the context of this specific case study. The first is that more than half of the immigrants were immigrants from FSU countries, which are characterized by high corruption indices. The most immigrants came from Russia and Ukraine, which ranked 154 and 105, respectively, in terms of corruption among 180 world countries, whereas Israel ranked 30, reflecting a low level of perceived corruption (Transparency International, 2025). This is in line with the study by Malézieux and Torgler (2021) on 13 different countries, which found that the tax morale of immigrants from low–tax-morale countries was lower than that of natives. Low perceptions of business ethics and high corruption in countries of origin might impact the tax morale of immigrants. Another possible explanation may be the disadvantaged position of immigrants in the new country, e.g., worse position in the labor market, working in jobs not commensurate with immigrants’ qualifications, discrimination, prejudice, social exclusion, etc.

The study identified two previously underexplored mediators, perceived dissatisfaction with tax–benefit reciprocity and national identification, that explain the relationship between immigrant status and tax morale in the investigated sample. Part of the relationship of being an immigrant on tax morale may be explained by the fact that immigrants feel less dissatisfied with the benefits received in return for tax payment than natives. This feeling reflects rational consideration for tax payment. Immigrants usually have lower incomes than natives (Kushnirovich, 2024), which means they pay lower taxes. Moreover, new immigrants in Israel are partially exempt from some taxes and enjoy special social insurance benefits in the initial years following migration. These exemptions are temporary and are gradually phased out. Lower taxes and enlarged social benefits may explain why immigrants are less dissatisfied with tax–benefit reciprocity. This finding is in line with other studies, which found that in Russia and several Asian countries, the tax morale of people who received smaller social benefits was lower (Alm et al., 2006; Torgler, 2004). The study also showed that lower dissatisfaction with received benefits vs. paid taxes was associated with higher tax morale. This aligns with the Reciprocity approach (Feld & Frey, 2007), which posits that when taxpayers feel the state provides adequate services in return for taxes, their intrinsic motivation to comply (tax morale) increases.

In sum, immigrants in the studied sample were less dissatisfied with tax–benefit reciprocity, and lower dissatisfaction was associated with higher tax morale, thereby increasing it. This partially buffered the negative relationship between identification and tax morale.

The study found that immigrants’ identification with the host country was lower than that of natives, and lower identification was associated with lower tax morale. This may be explained by a decreased perceived obligation and a lower sense of collective belonging and moral duty to contribute to the collective good, among individuals who identify themselves weakly with the country. Thus, the second mediator, identification with the country, heightened the negative association between immigrant status and tax morale.

The additional finding that income of investigated population is positively related to identification with the country is in line with Youngmann and Kushnirovich (2020), who found that in Israel income may be a resilience factor, which buffers and compensates for the negative effect of discrimination on immigrants’ well-being and improves immigrants’ feelings about the host country. Over time, the perceptions of immigrants converge with those of natives. The gap in tax–benefit reciprocity dissatisfaction between immigrants and natives closes after 10 years in the host country. One possible explanation is that immigrants may initially adopt system-justifying attitudes as a coping mechanism, which leads to higher satisfaction, but this erodes over time. This is in line with Jost et al. (2004), who noted that people, especially from disadvantaged groups, may defend and justify the existing social and institutional system to reduce cognitive dissonance. Over time, as they experience more inequality, this justification decreases. The gap in identification with the country disappears only after 25 years. Thus, the gap in rational considerations closes more quickly than the gap in social-psychological considerations. The gap in tax morale also closes after 25 years.

Because the sample under investigation was not representative, the findings of this empirical analysis could not be broadly generalized. Nevertheless, previous studies on tax morale using non-representative samples obtained similar findings. For example, Torgler (2004) in his experiment on tax morale used a sample comprising students and faculty members from two universities in Switzerland and Costa Rica; he found that tax morale depends on social norms in different cultural settings. The same findings were obtained by Cummings et al. (2009), who used in their experiments a non-representative sample recruited at universities, which was younger and more highly educated than the whole population, just as it was in the sample of the current study. The study of Bobek et al. (2013), which used a non-representative sample of 174 non-accounting students, found that social norms and perceptions of the tax system’s fairness, namely, fair reciprocity, correlated with tax compliance intentions. The study of Mickiewicz et al. (2019), which also used a non-representative sample, found that the sense of national identity positively predicts tax morale. Thus, other empirical studies using similar methods of sampling have found consistent results. Although there may be some criticisms, this study, based on a non-representative sample, is in line with earlier research in this field.

The study found that even after mediation, the association between being an immigrant and tax morale is significant. It means that other mediators can exist; further studies on them are needed. The study also found that high-income people have higher tax morale, and entrepreneurs have lower tax morale. Further studies should explain why these associations occur.

Even though immigration in Israel is based on ethnicity, recent studies have highlighted similarities in the economic and social integration of immigrants in Israel and other countries, debunking the myth of the uniqueness of Israeli immigration (Shuval & Leshem, 2017) and supporting the generalization of findings from Israeli studies to broader global contexts (Powell et al., 2017). Israel provides a theoretically informative case in which the coexistence of diverse immigrant groups (Chiswick et al., 2016; Semyonov et al., 2015), a strong state–citizen relationship, and recurring crises make the mechanisms of reciprocity dissatisfaction and identity-based belonging particularly salient. These mechanisms are not unique to Israel but correspond to universal motivational dimensions of tax morale that operate across societies.

The study had some limitations. The sample of immigrants is relatively small and non-representative; the analysis of a representative and larger sample may reveal additional associations. More than half of the immigrant respondents came from the FSU countries. The small numbers of immigrants from other countries, while reflecting the distribution of the immigrant population in Israel, did not allow for a comparison between immigrants from different countries of origin. The study’s cross-sectional design precludes any claims of causation and limits a thorough evaluation of mediation, despite the model’s strong theoretical foundations. Further experimental and longitudinal research is needed to investigate causality. Another limitation is that social-psychological motivation consists only of ‘identification with the country.’ Further studies are needed to investigate other dimensions of such motivation. Moreover, a single study based on data from a single country cannot provide strong evidence for the observed relationships; further studies are needed to support their existence as general phenomena.

The study also has some policy implications. It suggests that tax morale among immigrants can be improved by strengthening their identification with the host country and enhancing perceptions of fairness in the tax–benefit exchange. Tax authorities can apply service-oriented compliance measures, such as culturally competent helpdesks, simplified filing procedures, and proactive assistance, to lower administrative barriers that may otherwise undermine willingness to comply even when overall attitudes toward the system are positive. Practical implementation could also focus on early, targeted orientation and support for immigrants (e.g., multilingual information and services) that clearly explain tax obligations.

## 6. Conclusions

This empirical study examined how both tax–benefit reciprocity and identification with the country explain the tax morale of immigrants and natives in a single country. The results showed that immigrants generally have lower tax morale than natives, and this is partly explained by weaker identification with the host country. Higher immigrant satisfaction with received benefits vs. paid taxes buffers the negative association between identification with the country and tax morale. After 25 years of residence in the host country, the differences in tax morale between immigrants and natives largely disappear, suggesting a gradual process of adjustment and alignment with the norms of the host society.

## Figures and Tables

**Table 1:** Characteristics of the sample.

Characteristics	Native-Born Persons	Immigrants
Male, %	49.4%	55.8%
Age, years, Mean (SD)	33.9 (13.0)	38.3 (14.9)
Education, %	100%	100%
1 = less than high school;	0.8	2.0
2 = high school, no matriculation	6.2	2.0
3 = matriculation diploma	41.4	28.6
4 = vocational studies	6.9	8.2
5 = undergraduate degree	32.9	41.5
6 = graduate degree	10.3	14.3
7 = PhD, DSc., or other doctoral degree	1.5	3.4
Income, thousand ILS ^a^, Mean (SD)	11.924 (8.177)	11.344 (8.352)
Entrepreneur, %	25.7%	22.4%
Years residing in the host country:		
0–10		26.1%
11–25		35.2%
26 or more		38.7%

^a^ ILS = Israeli Shekel.

**Table 2:** Variables description.

Variable	Definition	Mean	(SD)
Tax morale	Based on 9 items scaled 1–5, from 1 = do not agree at all, to 5 = completely agree (Cronbach’s Alpha = 0.832):	3.37	(0.72)
	1.1. I understand and accept the reasons why people evade taxes (coded reversely)	3.02	(1.24)
	1.2. The self-employed would be bankrupt if they did not evade taxes (coded reversely)	2.83	(1.11)
	1.3. Tax evasion is the result of having no choice (coded reversely)	2.88	(1.17)
	1.4. Even law-abiding people evade taxes (coded reversely)	3.73	(1.05)
	1.5. Everyone who evades taxes is a criminal	2.05	(1.03)
	1.6. Those who evade taxes steal from other citizens of the country	2.23	(1.13)
	1.7. Tax evaders hurt us all	2.24	(1.09)
	1.8. The government loses billions because of tax evasion	2.19	(1.01)
	1.9. The government should be tougher on tax evasion	2.49	(1.03)
Feelings of dissatisfaction with the benefits received in return for tax payments	Based on the question “I feel that I pay a high tax compared to what the government provides me,” scaled 1–5, from 1 = do not agree at all, to 5 = completely agree	4.20	(0.94)
Identification with the country	Based on the question “To what extent do you feel your identity aligns with Israel”, scaled 1–5, from 1 = to a very small extent, to 5 = to a very high extent:	4.38	(0.87)

**Table 3:** Standardized effects of Model 4 (Mediation regression model in Process 4.2).

Independent Variables	Dependent Variables		
	Feelings of Dissatisfaction with the Benefits Received in Return for Tax Payments	Identification with the Country	Tax Morale
Being an immigrant	−0.239 *	−0.528 ***	−0.203 *
Gender	−0.063	−0.147 **	−0.011
Age	−0.062	0.087	0.215 ***
Income	0.177 **	0.111 *	0.154 **
Education	−0.161 **	0.018	0.007
Being an entrepreneur	−0.035	0.023	−0.130 **
Year	−0.045	0.037	−0.048
Feelings of dissatisfaction with the benefits received in return for tax payments			−0.118 *
Identification with the country			0.103 *

*** *p* < 0.001; ** *p* < 0.010; * *p* < 0.050.

**Table 4:** The differences in tax morale between immigrants by YSM and natives (ANOVA tests).

	Immigrants			Natives	Scheffe Tests
	0–10 Years Residing in Israel	11–25 Years Residing in Israel	26 or More Years Residing in Israel		
	(1)	(2)	(3)	(4)	
Tax morale, *Mean* (*SD*) ^a^ *F* = 20.120, *p* < 0.001	3.21 (0.21)	3.19 (0.22)	3.45 (0.27)	3.40 (0.23)	(1) vs. (2): *p* = 0.969 (1) vs. (3): *p* < 0.001 (1) vs. (4): *p* < 0.001 (2) vs. (3): *p* < 0.001 (2) vs. (4): *p* < 0.001 (3) vs. (4): *p* = 0.469
Feelings of dissatisfaction with the benefits received in return for tax payments, *Mean* (*SD*), *F* = 18.516, *p* < 0.001	3.16 (1.21)	4.42 (0.86)	4.20 (0.91)	4.29 (0.87)	(1) vs. (2): *p* < 0.001 (1) vs. (3): *p* < 0.001 (1) vs. (4): *p* < 0.001 (2) vs. (3): *p* = 0.670 (2) vs. (4): *p* = 0.823 (3) vs. (4): *p* = 0.922
Identification with the country, *Mean* (*SD*), *F* = 17.469, *p* < 0.001	3.54 (1.07)	4.06 (1.04)	4.44 (0.88)	4.50 (0.78)	(1) vs. (2): *p* = 0.045 (1) vs. (3): *p* < 0.001 (1) vs. (4): *p* < 0.001 (2) vs. (3): *p* = 0.156 (2) vs. (4): *p* = 0.007 (3) vs. (4): *p* = 0.962

^a^ Unstandardized predicted values.

## Data Availability

Restrictions apply to the availability of these data. Informed consent included the statement that the data would only be accessible to authorized project researchers.

## Appendix A

**Table A1:** Fit indices for CFA comparing one-, two-, and three-factor models.

Models	Model 1: One-Factor Model	Model 2: Two-Factor Model ^a^	Model 3: Three-Factor Model ^b^	Model 2 vs. Model 1 (∆)	Model 3 vs. Model 1 (∆)
*Χ^2^*	44.101	78.681	60.786	34.58	16.685
*df*	21	20	18	−1	−3
*CFI*	0.986	0.968	0.974	−0.018	−0.012
*NFI*	0.974	0.958	0.964	−0.016	−0.01
*IFI*	0.986	0.969	0.974	−0.017	−0.012
*TLI*	0.976	0.929	0.935	−0.047	−0.041
*RFI*	0.955	0.906	0.910	−0.049	−0.045
*RMSEA*	0.045	0.065	0.067	0.020	0.022
*AIC*	110.101	146.681	132.786	36.580	22.685
*BCC*	111.358	147.686	134.158	36.328	22.800

**^a^** Factor 1 (Normalization of tax evasion): I understand and accept the reasons why people evade taxes (coded reversely); The self-employed would be bankrupt if they did not evade taxes (coded reversely); Even law-abiding people evade taxes (coded reversely); Tax evasion is the result of having no choice (coded reversely). Factor 2 (Paying taxes is a duty): Everyone who evades taxes is a criminal; The government should be tougher on tax evasion; Those who evade taxes steal from other citizens of the country; Tax evaders hurt us all; The government loses billions because of tax evasion. **^b^** Factor 1 (Normalization of tax evasion): I understand and accept the reasons why people evade taxes (coded reversely); The self-employed would be bankrupt if they did not evade taxes (coded reversely); Even law-abiding people evade taxes (coded reversely); Tax evasion is the result of having no choice (coded reversely). Factor 2 (Tax evasion as a crime): Everyone who evades taxes is a criminal; The government should be tougher on tax evasion. Factor 3 (Tax evasion as harm to society): Those who evade taxes steal from other citizens of the country; Tax evaders hurt us all; The government loses billions because of tax evasion.
